# Current View in Platinum Drug Mechanisms of Peripheral Neurotoxicity

**DOI:** 10.3390/toxics3030304

**Published:** 2015-08-07

**Authors:** Alessia Chiorazzi, Sara Semperboni, Paola Marmiroli

**Affiliations:** 1 Experimental Neurology Unit and Milan Center for Neuroscience, Department of Surgery and Translational Medicine, University of Milano-Bicocca, Monza (MB) 20900, Italy; E-Mails: s.semperboni1@campus.unimib.it (S.S.); paola.marmiroli@unimib.it (P.M.); 2 PhD Program in Neuroscience, University of Milano-Bicocca, Monza (MB) 20900, Italy

**Keywords:** platinum drugs, peripheral neurotoxicity, mechanisms of action

## Abstract

Peripheral neurotoxicity is the dose-limiting factor for clinical use of platinum derivatives, a class of anticancer drugs which includes cisplatin, carboplatin, and oxaliplatin. In particular cisplatin and oxaliplatin induce a severe peripheral neurotoxicity while carboplatin is less neurotoxic. The mechanisms proposed to explain these drugs’ neurotoxicity are dorsal root ganglia alteration, oxidative stress involvement, and mitochondrial dysfunction. Oxaliplatin also causes an acute and reversible neuropathy, supposed to be due by transient dysfunction of the voltage-gated sodium channels of sensory neurons. Recent studies suggest that individual genetic variation may play a role in the pathogenesis of platinum drug neurotoxicity. Even though all these mechanisms have been investigated, the pathogenesis is far from clearly defined. In this review we will summarize the current knowledge and the most up-to-date hypotheses on the mechanisms of platinum drug-induced peripheral neurotoxicity.

## 1. Introduction

Since biological activity of cisplatin (CDDP) as anticancer drug was discovered in the 1970s and approved by FDA for the treatment of testicular and ovarian cancer in 1978, the use of this drug has become more diffuse overtime [1].

In the following years many other platinum drugs were synthesized and evaluated in clinical trials in order to overcome side effects and cases of CDDP resistance. Among them only carboplatin and oxaliplatin (OHP) are nowadays approved in all countries. Several other analogs of second and third generation are under study. CDDP is one of the most employed chemotherapeutic drug for testicular, ovarian, bladder, lung (small-cell and non-small-cell) cancer, sarcomas, solid tumors of head and neck. Carboplatin shows reduced side-effects but also less effectiveness as an anticancer drug if compared to CDDP. It is used in combination therapy as first line treatment of ovarian, lung, and breast cancer. OHP represents a cornerstone in the treatment of advanced and metastatic colorectal cancer (in combination with 5-fluorouracil/leucovorin); it is also used against digestive tract cancer [2,3,4].

One of the major side-effects limiting the clinical use of platinum drugs, as of several other anticancer drugs, is peripheral neurotoxicity, due to the fact that these drugs have easy access to the peripheral nervous system, which is less effectively protected from toxic substances compared to the central nervous system, where blood brain barrier is present. This represents a major clinical problem for the extensive use of these drugs in chemotherapy.

Several studies have been undertaken to investigate the physiopathology of platinum neurotoxicity. Many mechanistic hypotheses have been proposed but the pathogenesis is still to be elucidated.

In this review, the most up-to-date studies on the pathogenetic mechanisms of platinum drug peripheral neurotoxicity will be revised. In particular we will discuss the two main mechanisms recognized as primarily involved in the pathogenesis of platinum drug-induced peripheral neurotoxicity: the first is the formation of DNA adducts and crosslinks which damage DNA structure and lead to apoptosis; the second is the induction of oxidative stress and toxic effect on mitochondria as main events leading to neuronal apoptosis (mitotoxicity hypothesis).

Moreover, we will consider the issue of ion-channel involvement in the pathogenesis of the acute form of OHP neurotoxicity.

## 2. Platinum Drug General Toxicity

Platinum drugs are compounds containing metal ions which constitute binding sites for proteins, nucleic acids and other cellular molecules. This characteristic is in great part responsible for the biological activity of the drugs but also for their toxicity.

CDDP main general side effects are represented by nephrotoxicity, ototoxicity, severe nausea and vomiting, and mild hematologic toxicity. Nephrotoxicity is characterized by tubular damage and glomerular filtration impairment that can lead to severe damage to the kidney: this risk can be minimized through concomitant abundant hydration. Antiemetic drugs can acceptably reduce nausea and vomiting. Ototoxicity must be carefully monitored [5,6].

Carboplatin is a second generation platinum compound with hematological side effects (thrombocytopenia and anemia), which are dose dependent and represent the drug dose-limiting toxicity [5,6].

OHP is a third generation platinum drug. Among its major general toxicities are neutropenia, thrombocytopenia, nausea/vomiting, and diarrhea [5,7].

## 3. Platinum Drug Neurotoxicity

Platinum drug neurotoxicity induces the onset of a sensory neuropathy with symptoms involving all sensory modalities. All drugs are administered by intravenous infusion with variable dosage schedules, often in combination therapy.

CDDP produces peripheral neurotoxicity from mild to severe grade in most of adult patients after a cumulative dose in the range of 250–500 mg/m^2^, with disabling symptoms in about 10% of patients, representing a common dose-limiting problem. OHP causes symptoms of neuropathy in more than 80% of patients treated with a cumulative dose of 750–850 mg/m^2^; this toxicity may be severe in about 20% of the patients and be dose-limiting. Carboplatin is less neurotoxic at conventional doses even if peripheral neurotoxicity has been reported in a small percentage of patients also treated with taxanes [5,6,8,9].

In CDDP neurotoxicity we can observe symptoms and signs of an axonal neuropathy where damage of large myelinated sensory fibers is predominant. Patients initially complain of paresthesias and/or numbness in a stocking and glove distribution. On clinical examination, decreased distal vibration and proprioception with reduction/absence of deep tendon reflexes is observed. Other sensory modalities (pin, light touch, and temperature), though generally less compromised, may be involved. In patients with high grade neuropathy sensory ataxia may develop. Lhermitte’s sign (due to involvement of spinal cord dorsal columns) may be present [6,9,10].

Carboplatin neurotoxicity is less frequent and less severe. High doses of the drug or its use in combination therapy may produce a neuropathy similar to CDDP [2].

OHP induces two kinds of peripheral neurotoxicity, an acute form and a chronic one. With regard to the acute form, most of the patients treated with 85–130 mg/m^2^ develop a cold-induced clinical picture characterized by feet, hands, perioral paresthesias, and pharingolaryngeal dysesthesia; other less common but well described manifestations are jaw stiffness, cramps, shortness of breath, and difficulty in swallowing (rarely also pseudo-laryngospasm). These symptoms appear during infusion or in the first following hours and resolve within a week [8,11]. The chronic neuropathy has the same characteristics described in CDDP neurotoxicity.

In patients affected by platinum drug-induced neurotoxicity, neurophysiological examination shows a reduction in sensory action potential (SAP) while sensory conduction velocities could be normal or slightly decreased. No changes in motor nerve conduction and compound muscle action potential (CMAP) are usually reported [6,10]. On the other hand, electrophysiological studies shortly after OHP infusion evidence repetitive compound action potentials and high frequency discharges of motor units as in neuromyotonia, typical of excessive nerve excitability [6,8].

After discontinuation of platinum drugs, symptoms and signs of neuropathy may progress for 2–6 months. This effect is called “coasting phenomenon” and is relatively frequent. It mainly occurs after CDDP chemotherapy and is less evident with carboplatin [10].

Recovery from platinum drug neurotoxicity may be incomplete. In cisplatin-treated patients symptomatic or asymptomatic neuropathy is found in a considerable percentage of patients (10%–38%) evaluated many years after chemotherapy [10,12,13,14].

Even if some reports suggest that in the majority of patients OIPN (oxaliplatin-induced peripheral neurotoxicity) does not persist after chemotherapy discontinuation as a long term side effect, a recent study underlines that long-term follow-up performed with adequate neurological assessment evidenced persistent symptoms and signs of neuropathy in nearly 80% of oxaliplatin-treated patients at a median of 25 months after the end of OHP treatment [14]. It is conceivable that multimodal clinical assessment (neurological examination, scales, questionnaires, neurophysiological assessment) constitute a better instrument to evaluate persistence of neuropathy [15].

A systematic review of OIPN literature concluded that abnormalities in nerve function persist in a variable but significant number of patients after at least 12 months from the end of OHP administration [16].

Other studies with a follow-up of 5–6 years reported persistence of OIPN in almost 35% of patients [17,18]. The influence of a higher cumulative dose in the development of long-term platinum drug-induced neuropathy has been proposed but not completely demonstrated.

## 4. Main mechanisms of Platinum Drug Neurotoxicity

As already stated in the introduction, we will discuss the two main mechanisms of platinum drug neurotoxicity in detail: the DNA structure damage and the oxidative stress/mitotoxicity hypothesis.

With regard to the acute form of OHP toxicity we will review the current knowledge on the role of ion channels and calcium signaling.

### 4.1. Nuclear DNA Damage

Metal based molecules are biologically active due to their binding with important biological entities such as proteins, enzymes, hormones, and nucleic acids. Probably this is related to the presence of vacant d-orbitals and to the positive charge on metal ions, which act as binding sites [3,19]. Even if DNA is not the exclusive target of these compounds [20], the cytotoxicity of platinum anticancer drugs essentially depends on their ability to interact with DNA, forming DNA adducts, intra-strand, and inter-strand DNA cross-links and also DNA-protein cross-links. These alterations are able to stop DNA replication and cell cycle, to inhibit DNA repair mechanisms and to induce cell death through apoptosis [21,22]. The peculiar biological activity of these platinum drugs is also responsible for their side effects, including neurotoxicity.

CDDP, carboplatin and OHP share the same mechanism of action. PlatinumII atom center, produced by platinum drug hydrolysis, is responsible for platinum drug interaction with various molecular targets. This molecule is a soft acid and it preferentially reacts with soft, easily polarizable bases, forming covalent bonds with appropriate nucleophiles, in particular sulphur-containing nucleophiles over amine *N*-nucleophiles and *O*-nucleophiles [20].

The formation of DNA-Pt adducts (mainly in the S-phase of the cell cycle) distorts the DNA double helix structure, thus preventing DNA replication and causing an inhibition of proliferation, in fact the cell cycle arrests in G2/M phase. Studies conducted using X-ray crystallography and MRI have solved the structure of these adducts showing that these cross-links lead to the formation of abnormal bending and to the unwinding of the DNA double helix [23,24]. These alterations explain the platinum drugs’ ability to prevent DNA synthesis.

It has been shown that the CDDP in mono-hydrate form preferentially binds to the purine imidazole ring, guanosine (N7 atom) and adenosine (N1 or N7 atoms). If after an initial binding to purines there is another potentially reactive site nearby, another reaction could take place, leading to cross-links formation between strands and intra-strand. *In vitro* studies have shown that CDDP treated DNA mainly contains intra-strand cross-links of 1,3-d(GpNpG) type and 1,2-d(GpG) type, the latter being considered the main responsible for the drug cytotoxic action [25]. There is also the formation of a small percentage of inter-strand cross-links and monofunctional adducts.

Cross-links between guanine bases are also induced by carboplatin and OHP, leading to the same consequences. Carboplatin has been considered as a pro-drug for CDDP and it has a slower rate of conversion to reactive species compared to CDDP. This compound causes the same cross-links as CDDP, which is consistent with the knowledge that CDDP-resistant tumors are cross-resistant to carboplatin [26]. The main carboplatin reaction path involves the direct attack by nucleophiles *via* ring opening and subsequent binding with DNA [3,27]. However this drug is well-tolerated by patients and has fewer side effects, so it can be given in higher doses compared to CDDP.

On the other hand OHP causes structurally different adducts [26]. In fact it mainly forms GpG intra-strand adducts with bulky hydrophobic diaminocyclohexane (DACH) ligand putting a major groove into DNA [28]. The DNA-DACH-Pt adducts are bulkier and more hydrophobic than CDDP formed adducts, yet OHP is more cytotoxic than CDDP [29,30].

In general, for platinum drugs, biochemical analyses demonstrate that 1,2-intra-strand AG and GG cross-links account for about 85%–90% of all DNA adducts [31]; in contrast, the 1,3-interstrand (GpG) cross-links and monofunctional adducts make up about 2%–6% of the platinum bound to DNA each [26].

In the nervous system, the main target of platinum drugs are dorsal root ganglia (DRG) [32], where morphological alterations in the nucleolus may be seen and DNA damage is thought to be the main cause of apoptosis observed in sensory neurons [33]. Platinum-based agents have the propensity to enter the DRG because they lack a blood brain barrier and are vascularized by fenestrated capillaries that make them more accessible to circulating compounds, including exogenous toxic substances as platinum. Once entered in the DRG, the platinum-based compounds form DNA-Pt adducts causing apoptosis. Although DRG neurons are postmitotic and non-dividing cells and DNA-Pt adducts are not lethal, the amount of these accumulated adducts is correlated to neurotoxicity severity [5]. Platinum adducts probably cause axonal changes secondary to neuronal damage.

OHP and CDDP differ in neurotoxicity severity in the DRG. In fact, CDDP causes DNA-Pt adduct formation about three times more frequently than OHP [30]. This confirms the hypothesis of a correlation between DNA cross-links levels induced by platinum and peripheral neuropathy severity induced by platinum drugs. Furthermore differences in neurotoxicity degree could also be explained by dissimilar plasma concentrations of the intermediary products of the aquation to DNA adduct formation process [34]. CDDP and OHP undergo hydrolysis to a greater extent than carboplatin, which may contribute to the difference in the associated neurotoxicity severity patterns [5].

As already stated, platinum drug cytotoxicity depends on the interaction with DNA and final induction of apoptosis. The activation of this process happens as a consequence of the recognition of such DNA alterations from proteins deputed to detect DNA damages. Actually the proteins involved in DNA repair pathways play a pivotal role in modulating the drugs cytotoxicity. The Nucleotide Excision Repair (NER) pathway is a repair system able to fix a wide spectrum of lesions presenting the distortion of the DNA helix. Then NER represents the main recognition mechanism and possible correction of DNA-Pt adducts. The DNA lesions repair performed by NER involves several steps: after the recognition of DNA-Pt adduct, a helicase opens the DNA helix and then a specific endonuclease removes a segment of about 30 bases containing the lesion. Finally a new strand is synthesized using the intact strand as a template [35]. Although NER recognizes all types of intra-strand cross-links (1,2-d(ApG), 1,2-d(GpG), 1,3-d(GpNpG)), the intra-strand cross-links 1,2 are less efficiently repaired, supporting the hypothesis that these latter lesions are the most cytotoxic. Cells from the peripheral nervous system are not able to efficiently remove DNA lesions induced by platinum due to an inefficient NER pathway. The DNA-Pt adducts not removed from NER do not allow ribosomal RNA correct transcription, causing an incomplete protein synthesis. DRG neurons are cells with high metabolic activity and so the lack of physiological dense ribosomal RNA synthesis could be lethal for this cell type [36]. Also Nouspikel and Hanawalt (2002) reported a general downregulation of several DNA repair mechanisms, including NER, in terminally differentiated neuronal cells [37]. The inefficient repair mechanism system could be responsible for apoptosis induction in DRG neurons. In 1998, Gill supposed that CDDP cytotoxic action in neurons was due to its attempt to re-entry into the cell cycle [38]. However the DRG neurons are unable to complete DNA replication, probably because the neuronal DNA has been crippled by accumulated lesions due to the absence of an efficient repair pathway, and they die shortly thereafter. Confirming this hypothesis, an increased expression of genes involved in cell cycle regulation and apoptosis was observed also by Alaedini and colleagues (2008) through microarray analysis in DRG of rats after treatment with CDDP. In particular, overexpression of the gene for cyclin D1, p21, and BID3 was reported [39].

NER also mediates the repair of nucleosomic DNA lesions induced by platinum compounds. The Pt-adducts with nucleosomic DNA are able to block the access to DNA template by RNA polymerase; in 2003 Wang and colleagues observed that, if the formation of DNA-Pt adducts involves the nucleosomes, NER cannot repair them, as its activity is completely inhibited by these adducts [40]. Since the nucleosomes play a key role in modulating many cellular processes, including recombination, replication, transcription, and DNA repair, their alteration has strong consequences on the entire cell.

So NER plays a dual role in modulating the CDDP cytotoxicity: although its inability to remove the DNA-Pt adducts from nucleosomes and to repair intra-strand cross-links 1,2 is the basis of the cytotoxic effect of platinum drugs, on the other hand an increasing expression of genes encoding proteins involved in this repair mechanism may make it more effective, causing the onset of drug resistance [21]. This is the case for example of the two genes *Xeroderma Pigmentosum A* (*XPA*) and *Excision Repair Cross-Complementating 1* (*ERCC1*), that encode two essential key enzymes for the NER repair process. Their expression level is predictive of OHP sensitivity in six colon cell lines *in vitro* [41]. Increased activity of ERCC1 and XPA also correlates with CDDP resistance. On the other hand the XPA proteins functional loss prevents the DNA-Pt adducts removal, so that they accumulate, increasing DNA damage [36]. This is demonstrated in testicular cancer cells: they are hypersensitive to CDDP treatment since they are defective in XPA proteins [42].

DNA-Pt adducts represent recognition binding site for other cellular proteins, such as transcription factors, histones, and High Mobility Group (HMG) domain proteins [24]. More than 20 different proteins able to detect conformational change in DNA structure induced by DNA intra or inter-strand cross-links caused by platinum have been described. Each of these proteins is able to specifically detect a different kind of DNA-Pt adducts, triggering a specific molecular cascade that leads to cell death. So the specificity of each protein in recognizing bendings and unwindings of the DNA double helix may be at the basis of the different mechanisms of action of platinum-based chemotherapeutic drugs [26].

For example, the mismatch repair (MMR) complex proteins are able to recognize and then to tie the DNA cross-links induced by CDDP with greater affinity than those induced by OHP and carboplatin, according to the evidence that MMR seems to be crucial for the sensitivity to CDDP, while not involved in the mechanism of OHP or carboplatin-induced cytotoxicity [43]. The HMG domain proteins represent more than 70% of all the proteins that interact with 1,2 intra-strand cross-links induced by CDDP [44], making the DNA filament inaccessible to the enzymes deputed to the genetic code transcription and repair [45,46].

Platinum drugs exert their cytotoxicity also altering the binding of transcription factors to their recognition sites and blocking protein synthesis. For example some transcription factors required for recognition of the promoter by RNA polymerase manifest a higher affinity of binding to DNA complexes with CDDP and the transcription factor TATA box binding protein (TBP) binds selectively to DNA damaged by CDDP. So the drug subtracts TBP to its physiological role, inhibiting the transcription [47,48].

### 4.2. Mitochondrial DNA Damage and Oxidative Stress

Mitochondrial DNA (mtDNA) is a circular 1600 pb piece of DNA that encodes for 13 proteins important for the production of the complex involved in the electron transport chain and for 22 tRNA and 2 rRNA essential for the production of these proteins [33,49].

While it is well known that the platinum compounds bind to nuclear DNA inducing apoptosis, recent studies showed that these compounds bind also the mtDNA inducing myotoxicity. Several animal models evidenced that the treatment with antineoplastic agents leads to the onset of myotoxicity. Platinum drugs induce adducts with mitochondrial DNA (mtDNA-Pt adducts), randomly around the mitochondrial genome, and these adducts cause the same alterations shown for nuclear DNA such as the disruption of mtDNA replication and transcription and morphological changes within the mitochondria. Pondratz and colleagues (2011) observed in DRG neurons treated with CDDP that the transcription complex disengages when it meets mtDNA-Pt adducts and that the transcription machinery is constantly trying to reengage on the mitochondrial genome [49]. These mtDNA fragments are incomplete and for this reason dysfunctional and destined to degradation. Morphological abnormalities in mitochondria reflect mitochondrial dysfunction due to deficiency in mtDNA [50]. It has been demonstrated that, at mitochondrial level, NER does not work and consequently DNA lesions accumulate instead of being removed. The final result of this damage induces chronic energy deficit with degeneration in sensory primary afferent neurons and chronic peripheral neuropathy [51,52].

In the peripheral nervous system 95% of the mitochondria are localized in the axons, so mitochondrial dysfunction likely causes degeneration of the axonal transport, a process that requires energy [33]. These changes may be predisposing factors also for the onset of neuropathic pain [52,53]. Probably the alteration of mitochondrial function is also at the basis of the coasting phenomenon, whose symptoms may progress several months after discontinuation of platinum drug administration [2].

Platinum drug-induced myotoxicity often leads to an increase in reactive oxygen species (ROS) that results in the onset of oxidative stress responsible for neuronal damage [54]. It is known that, under oxidative stress conditions, ROS increase can cause alteration of cellular protein, lipid and DNA inducing cell damage and mitochondrial malfunction [4,55]. The onset of oxidative stress is involved in CDDP toxicity. Several studies showed that the excessive production of ROS induced by CDDP treatment can lead to apoptosis through extrinsic and intrinsic pathways and can induce cell death through autophagy [4,56,57].

It is known that the oxidative equilibrium is strictly related to the onset of pain and that not only mitochondria but also peroxisomes are involved to maintain the redox cellular state [58] considering that they play a role in production and scavenging of ROS [59].

Mitochondrial damage and oxidative stress have been reported in many studies dealing with toxicity mechanisms of CDDP and/or OHP and carboplatin [4,51,52,60] while the involvement of peroxisomes are not widely studied [58].

From literature it is known that, in a milieu with low concentration of chloride, the chloride ions of CDDP are replaced by water and this complex can cause the increase in ROS levels [61,62,63]. The *in vitro* and *in vivo* treatment with CDDP showed an increase in ROS levels that leads to an increase in lipid peroxidation, a reduction in catalase activities and glutathione peroxidase, an over-activation of caspase 3/7, apoptosis, and DNA fragmentation [64,65]. Melli and colleagues used an *in vitro* study to investigate if the toxicity induced by CDDP on DRG neurons was related to mitochondrial loss of function and increase in oxidative stress. They observed that DRG neurons showed a decrease in mitochondrial function, a loss of membrane potential and mitochondrial structural changes [64,66].

In 2009 Custodito and colleagues performed an *in vivo* study in which they observed that the treatment with CDDP in rats is able to induce changes on mitochondrial membrane ion permeability in liver [67]. Podratz and colleagues demonstrated in 2011 that CDDP is able to bind mtDNA in DRG neurons at a similar rate as nuclear DNA. This binding with mtDNA leads to alteration of mtDNA replication and transcription and is able to induce morphological changes in mitochondria [49]. They also demonstrated that mtDNA-Pt adducts could produce energy failure and prolong the toxicity after CDDP removal.

These and other several models suggest the hypothesis that mitochondria dysfunction and mitochondrial membrane potential reduction could be important in the pathogenesis of neurodegeneration in CDDP-related peripheral neuropathy [4,64,68]. Already in 1990 Mollman and collaborators suggested an important involvement of oxidative stress in the onset of neurotoxicity induced by CDDP. In their study they observed that the CDDP neuropathy is clinically similar to the neuropathy observed in patients with deficiency in vitamin E that is a molecule useful to detoxify the ROS [69].

Recent works demonstrated that OHP treatment in rats is able to induce an increase in superoxide anion production, lipid peroxidation and protein and DNA oxidation, and that the increase in superoxide anion production is not related to oxidative property of OHP but could be dependent on mitochondrial damage [70,71]. Zheng and collaborators in 2011 also showed that OHP administration produces effects on axonal mitochondria and consequent disruption of electron transport chain and cellular energy failure in DRG neurons [71]. These mitochondrial changes could be the cause of the increased ROS generation. Also Nassini and colleagues in 2011 observed a relationship between OHP-induced neurotoxicity and oxidative stress [72].

Zanardelli and colleagues [58] evaluated in an *in vitro* and *in vivo* study the role of peroxisomes, which are known to be involved in chronic pain [73,74], in OHP-induced neurotoxicity. In their experiments, they observed an increase in peroxisomes number after OHP treatment, although expression and functionality of catalase were reduced in DRG and spinal cord. These results suggest a relationship between oxidative damage and neuropathic pain in the model used in this study.

Some experiments showed a possible role of Transient Receptor Potential Cation Channel 1 (TRPA1) in mediating hypersensitivity to cold provoked by OHP *via* oxidative stress-related events [33,72,75]. Joseph and colleagues in 2008 and Di Cesare Mannelli in 2012 showed that the co-treatment with OHP and a natural antioxidant compound is able to prevent oxidative phenomena and to decrease OHP-dependent hyperalgesia and allodynia in rats [76,77].

A recent study [78] evidenced that mice co-treated with OHP and mangafodipir (a drug with SOD-, catalase and glutathione reductase-like properties) had less severe neuropathy induced by the OHP treatment, and this result was confirmed in a small series of patients.

Cheng and colleagues demonstrated that the treatment with carboplatin in mice induces increased levels of caspase 3/9, cytocrome-c and ROS and decreased levels of anti-apoptotic proteins. These observations suggest that carboplatin determines its toxicity *via* a mitochondrial pathway related to increased oxidative stress [60]. It has also been reported that oxidative stress induced by the treatment with carboplatin causes tissue injury and ROS levels increase, implicated in several cardiovascular diseases such as hypertension, atherosclerosis, and heart failure [60,79].

### 4.3. Ion Channels and Role of Calcium Signaling

The pathogenetic hypothesis concerning ion channels and calcium signaling will be specifically considered in relationship to acute OHP neurotoxicity.

OHP, compared with other platinum drugs, has a unique characteristic that is the acute form of neurotoxicity it provokes. This is a very frequent but rapidly reversible dysfunction, during which patients experience symptoms due to axonal hyperexcitability as confirmed by neurophysiological examination.

The most accepted hypothesis explaining the clinical picture is dysregulation of nodal voltage-gated Na^+^ channels with alteration of Ca^2+^ signaling.

It is believed that the OHP metabolite oxalate could determine a rapid chelation of calcium (Ca^2+^) and magnesium (Mg^2+^) responsible for alteration in voltage-gate sodium channel kinetics resulting in axonal membrane hyperexcitability [33,53].

However, the mechanisms at the basis of ion channel dysfunction are not sufficiently clarified.

A recent study in patients with colorectal cancer [80] compared transient and persistent Na^+^ conductance before and after a single OHP infusion, using specialized neurophysiological examination. It concluded that acute OHP administration causes changes only in transient Na^+^ conductance.

The study of Grolleau *et al.* (2001) was one of the first work indicating a role of voltage-gated sodium channels in acute OHP neurotoxicity [81]. Intracellular registrations of neurons isolated from the central nervous system of the cockroach were performed and changes induced by OHP intracellular application on sodium current were examined. They demonstrated that OHP was able to lower the amplitude of the action potential by reducing the voltage-dependent sodium current. Moreover authors suggested that this change could also involve calcium ions through the chelating action of OHP metabolite oxalate.

A reduction in the peak amplitude of voltage-gated sodium current was also reported by Wu *et al.* [82], who studied the effect of OHP on ion currents in NG108-15 neuronal cells (motor-neuron-like cell line). In addition, a delay in the inactivation time of voltage-gated sodium current was demonstrated. In this work the same results were obtained when HEK293T (human embryonic kidney) cells transfected with SCN5A (that lead to the appearance of voltage-gated sodium current) were exposed to OHP [82]. In authors’ opinion OHP is likely to act blocking the open channel state; the consequent slowing of current inactivation could be a mechanism at the basis of OHP acute peripheral neurotoxicity [82].

Recent studies suggested a specific role of sodium channel Nav1.6, one of the main nodal sodium channels in peripheral myelinated axons, in OHP acute form of neurotoxicity. Results from axonal and DRG neuron recordings evidenced that this channel subtype is implicated in mediating enhancement of persistent and resurgent sodium current induced by OHP and cooling [83].

Also Deuis *et al*. using a new mouse model of acute OHP induced peripheral neuropathy in which the drug is administered by intraplantar injection, suggested a crucial role of sodium channel Nav1.6 in the development of cold allodynia in experiments with selective channel inhibitors and knock-out (KO) animals [84].

Recent genetic studies which identified single nucleotide polymorphisms of voltage-gated sodium channels genes (*i.e.*, *SCN4A-rs2302237* and *SCN10A-rs1263292*) being significantly associated with higher frequency of acute OHP-induced neuropathy support the hypothesis of a central role of sodium channels in the pathogenesis of this kind of neurotoxicity [53,85].

Although literature data suggest a major involvement of sodium channels in the pathogenesis of acute OHP neurotoxicity, there are also indications of a possible role of voltage-gated potassium channels [86]. Kagiava *et al*. in *in vitro* experiments testing the effect of OHP on evoked compound action potential of isolated rat sciatic nerve, found malfunction of potassium, but not of sodium, channels [87].

Deuis *et al.*, who suggested a crucial role of sodium channel Nav1.6 in acute OHP peripheral neuropathy, also showed in the same study that OHP acts as inhibitor of neuronal potassium channels leading to increased excitability [84].

Moreover some authors, studying the hypersensitivity to cold typical of acute OHP neurotoxicity, demonstrated that this drug modulates potassium ion channels involved in thermal sensation. In particular, OHP infusion was found to downregulate potassium channels TREK1, TRAAK, and upregulate hyperpolarization-activated HCN1 channels, TRPA1, and Na_v_1.8 in DRG. Interestingly the data were supported by the observation that, in TREK1-TRAAK KO mice and using the HCN inhibitor ivabradine, hypersensitivity to cold was respectively abolished or reduced [88].

As far as calcium signaling is concerned, a number of findings indicates that it could be involved in the pathogenesis of various chemotherapeutic drugs neurotoxicity, as OHP, taxanes, vinca alkaloids [89,90]. Schulze *et al.* [90] reported that acute OHP exposure does not interfere with calcium signaling but, on the contrary, prolonged OHP exposure determines a sensitizing effect on intracellular calcium. In a recent work Kawashiri *et al.* [91] demonstrated that in DRG neurons primary cultures OHP administration induced a dose dependent increase in intracellular calcium and that some calcium channel blockers could revert this effect. Moreover, in a retrospective clinical study on patients treated with modified FOLFOX6 therapy, a subgroup of patients pre-treated also with calcium channel blockers had a significantly lower cumulative incidence of acute neuropathy. On the other hand, no differences regarding the incidence of chronic neuropathy were found between groups [92].

Results of several recent randomized clinical trials testing the effectiveness of calcium/magnesium infusion to counteract acute OHP neurotoxicity are available [93,94,95].

Despite a theoretical basis for the use of this kind of neuroprotection, the overall results and in particular the two most recent trials [94,95] failed to demonstrate a real protection of calcium/magnesium infusion in acute OHP neurotoxicity.

The role of ion channels modification and Ca signaling is mainly related to the acute form of OHP neurotoxicity. As acute neurotoxicity is almost never the cause of treatment discontinuation, mechanistic studies are generally more concentrated on the chronic form, which is often dose-limiting or long lasting. However, understanding the pathogenesis of acute OHP neurotoxicity must not be considered a secondary aim as a relation with the severity of the chronic form has been suggested. A proved relationship between the two kinds of neurotoxicity has not been found yet but it has been reported that patients who have more complicated acute symptoms often show a more severe type of chronic neurotoxicity [11]. Moreover, the hypothesis of, at least in part, a common genetic susceptibility has been advanced by some authors and is under investigation [53].

## 5. Conclusions

Peripheral nervous system impairment represents a major dose-limiting factor in platinum drug chemotherapy.

In this review we describe the main pathogenetic hypotheses on platinum drug-induced peripheral neurotoxicity, but extensive investigations are still necessary to clarify its pathophysiology and discover effective preventive treatments. In fact, even if some neuroprotective compounds seem to be promising, no agent can be definitely recommended for the prevention and treatment of neurotoxicity due to platinum-based therapy so far.

Moreover, in patients the course and severity of platinum drug-induced peripheral neurotoxicity is variable, so a genetic predisposition has been suggested. Indeed, the identification of polymorphisms in genes involved in the biological activity of platinum drugs might help in explaining differences in the severity of the nervous system damage. Individual genetic variation in the drug toxicity response might be used as a clue to direct future mechanistic experiments.
